# Patient compliance with braces vs. Invisalign^®^: a secondary data analysis from a randomized clinical trial

**DOI:** 10.1590/1678-7757-2025-0179

**Published:** 2025-08-11

**Authors:** Graziela Hernandes VOLPATO, Paula Vanessa Pedron OLTRAMARI, Renata Rodrigues de ALMEIDA-PEDRIN, Thais Maria Freire FERNANDES, Marcio Rodrigues de ALMEIDA, Gabriela UTRAGO, João POIANI, Ana Cláudia de Castro Ferreira CONTI

**Affiliations:** 1 Universidade do Norte do Paraná Departamento de Ortodontia Londrina Paraná Brasil Universidade do Norte do Paraná, Departamento de Ortodontia, Londrina, Paraná, Brasil.; 2 Universidade Anhanguera (UNIDERP) Departamento de Ortodontia Campo Grande Mato Grosso do Sul Brasil Universidade Anhanguera (UNIDERP) - Departamento de Ortodontia, Campo Grande, Mato Grosso do Sul, Brasil.; 3 Universidade de São Paulo Faculdade de Odontologia de Bauru Departamento de Ortodontia São Paulo SP Brasil Universidade de São Paulo, Faculdade de Odontologia de Bauru, Departamento de Ortodontia, São Paulo, SP, Brasil.

**Keywords:** Orthodontics, Aligners, Compliance

## Abstract

The success of orthodontic treatment depends on several factors that may influence the desired outcome, such as patient motivation and cooperation. Objective: This study compared patient compliance during the first year of orthodontic treatment using aligners and conventional fixed appliances. Methodology: The sample included 39 participants (22.1±4.62 years, 25 males, 14 females) randomized into two groups: orthodontic aligners (OA, 20 patients, 23.7±5.6 years, 12 males, 8 females) and fixed appliances (FA, 19 patients, 20.5±4.5 years, 12 males, 7 females). Patient compliance was measured using the Orthodontic Patient Cooperation Scale (OPCS) composed of 10 questions related to attitudes and attendance of patients in relation to treatment. The questionnaire was applied at three different points: at 3 months (T1), at 6 months (T2), and at 12 months (T3) of treatment. The psychosocial profile of the patients was assessed using the anxiety (IDATE) and stress (PSS-14) questionnaires. Both groups were comparable in age, gender and psychosocial profile. Compliance comparisons between groups at T1, T2 and T3, as well as between genders, were performed using the Mann-Whitney test. Comparison between T1, T2 and T3 were performed using the Friedman test. The correlation between age and compliance scores was assessed using the Spearman's correlation coefficient. The psychosocial profile was analyzed using the Independent t-test. All tests considered p<0.05. Results: The type of appliance (OA or FA) and the time of assessment did not significantly influence patient compliance. The age and gender of patients were not correlated with their degree of compliance. Patient compliance was similar in the first 12 months of treatment, regardless of the protocol used, the patient's gender and age. Conclusions: The level of patient compliance with orthodontic treatment was not influenced by the type of appliance (conventional fixed appliances or aligners), nor by the patient's age or gender.

**Figure:**
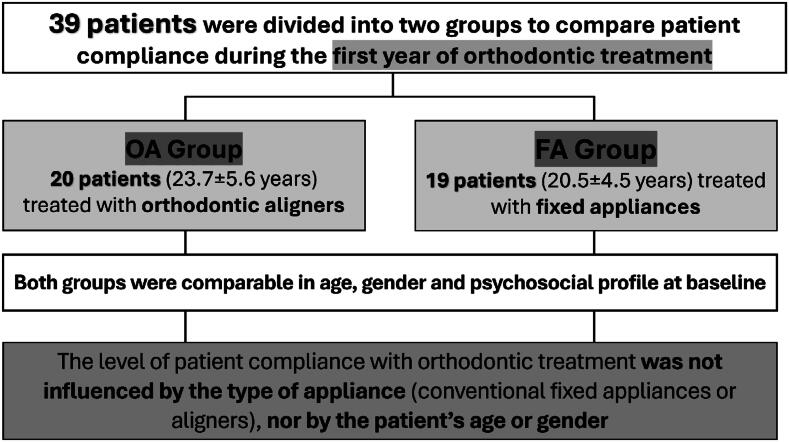


## Introduction

The success of orthodontic treatment is related to several factors that may influence the desired outcome.^1 - 3^ Motivation and cooperation in treatment adherence have guided some clinical studies.^1 , 2 , 4 - 6^ Patient compliance with fixed and removable appliances is essential for achieving established objectives.^7 , 8^ Adequate oral hygiene and the maintenance of intermaxillary elastic or removable devices to prevent daily wear are important factors to consider.^4 , 5 , 7 , 9^ The patient needs to be self-disciplined and dedicated in following professional instructions to achieve the desired treatment results.^10^

Fixed braces have been the conventional and effective orthodontic appliance for over a hundred years.^11^ However, in recent years, there is a growing demand for esthetic and comfortable orthodontic treatment among adults and adolescents.^11 , 12^ Aligner systems have shown their great efficacy in correcting various type of malocclusion, such as crowding, misalignment and diastemas.^13 - 16^ A previous study has demonstrated that bodily tooth movements, including molar distalization, incisor torque, and premolar derotation, can be achieved using the Invisalign^®^ system.^16^ Case reports have shown that aligners can be used in cases involving extractions and poor occlusal relationships.^14 , 15^ However, more extensive research evaluating clear aligner treatment for treating complex malocclusions is still needed.^13^

Despite the great esthetic appeal of these devices,^17 - 19^ aligners constitute a removable device and clinicians should consider the characteristics of these two orthodontic appliances when making treatment decisions.^11 , 20 , 21^ Clear aligners have advantages in segmented tooth movement and shortening treatment duration.^11^ However, a recent systematic review reported no significant difference in treatment duration between fixed appliances and clear aligners, suggesting that further research is needed to clarify this point.^13^ Braces are more effective in achieving adequate occlusal contacts, controlling tooth torque, increasing transverse width and providing better retention.^11^

Previous studies have assessed the effectiveness and satisfaction with aligners.^18 , 20 - 23^ The pain intensity and the incidence of external apical root resorption associated with these two types of appliances have been previously studied.^24 - 27^ However, the degree of patient compliance must still be investigated.^11 , 20^ No research was found in the literature comparing patient cooperation when using aligners and fixed appliances. The aim of this study was to verify the degree of patient compliance when using aligners versus conventional fixed appliances during the first 12 months of orthodontic treatment. The null hypothesis was that there is no difference in patient compliance between using aligners and fixed appliances during the first 12 months of treatment.

## Methodology

### Trial design

This study was a secondary data analysis based on a randomized clinical trial.^27^ Participants were prospectively recruited and randomly allocated into two groups. No changes in the methods occurred after the trial began.

### Participants, eligibility criteria and settings

The sample was obtained by screening 2,662 individuals assessed via social media and on schools in the city of Londrina/PR, Brazil.

Participants who met the following criteria were included: Angle Class I malocclusion, moderate crowding and treatment without extraction. The following characteristics were considered as exclusion criteria: absence of permanent teeth, anterior or posterior open and cross bite, dental anomalies, and previous history of orthodontic treatment.

The research was approved by the Institutional Review Board of University of North Paraná (UNOPAR) (CAAE: 12088219000000108) and registered in Brazilian Clinical Trials (ReBEC: 9zytwf). Volunteers received treatment at a graduate clinic and were assisted by orthodontists supervised by an orthodontics professor with 15 years of experience.

### Interventions

All patients underwent initial orthodontic records including intra and extraoral photographs, digital dental models and radiographs.

Patients were randomly allocated into two groups, as follows:

– Orthodontic aligners (OA) (Smart Track, Invisalign^TM^, Align Technology): Virtual planning was applied for this group (ClinCheck^TM^ Pro program, version 5.6, Align Technology). The pairs of upper and lower OAs were changed at every 10 days, with a recommended daily use of 22 hours.
– Fixed metallic orthodontic appliance (FA) (slot 0.022 × 0.030", 3M Unitek, Monrovia, California, USA): These patients had appliances attached to all teeth and the same sequence of archwires (superelastic nitinol 0.014", 0.016" and 0.016 × 0.022") was used in the first 6 months of treatment. After that period, the archwires were individualized according to each patient's needs.

For both groups, monitoring procedures were performed monthly.

### Measurement of the degree of patient compliance

**Figure 1:**
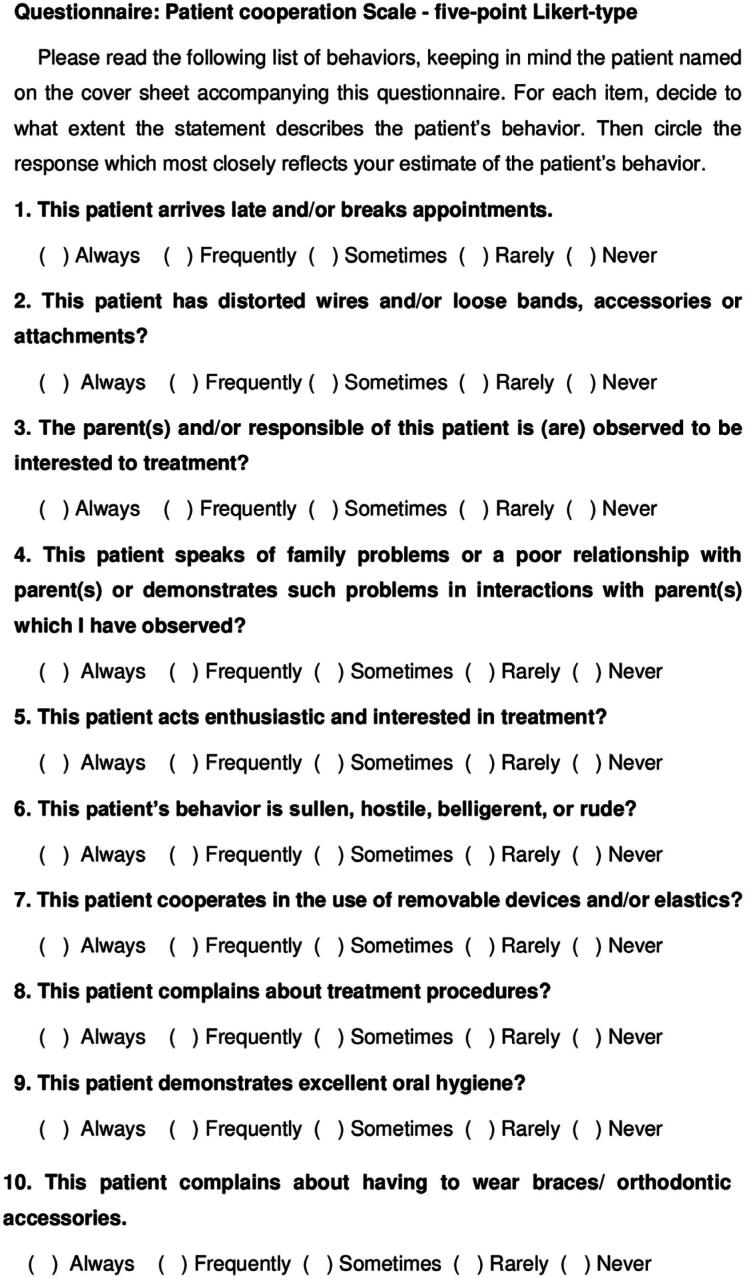
Questionnaire: patient compliance Scale—five-point Likert-type

The degree of patient compliance was measured using the Orthodontic Patient Cooperation Scale (OPCS) revised and validated by Slaker, et al.^28^ (1980). This scale consisted of 10 questions regarding patients’ attitudes and appointment attendance with Likert answer options ( Figure 1 ). The questionnaire underwent the following adaptations: The sentence "Accessories or attachments" was added in question two; the word "headgear" was replaced by "removable devices" in question seven. The questions were always answered by two orthodontists participating in the research, who performed a previous calibration of how to fill out the questionnaire in the three different assessment periods: after 3 months (T1), 6 months (T2) and 12 months (T3) of treatment. The validation of the questionnaire in Portuguese was performed using alpha statistics.^29^

A quantitative score was assigned to each five-choice Likert qualitative response, (never = 1, rarely = 2, sometimes = 3, often = 4, always = 5). For negative questions (1, 2, 4, 6, 8, 10), the score was inverted (never = 5, rarely = 4, sometimes = 3, often = 2, always = 1). The sum of each patient's 10 behaviors reached scores ranging from 10 (lowest compliance) to 50 (highest compliance).

### Psychosocial profile of patients

Psychosocial variables that could impact the profile of patients, such as anxiety and stress, were evaluated at baseline (T0) using the State-Trait Anxiety Inventory (STAI-T) and the Perceived Stress Scale (PSS-14), respectively.

### State-Trait Anxiety Inventory (IDATE)

The STAI-T has 20 items with scores ranging from one to four (1-not at all, 2- a little, 3- moderately, 4- very much). The score varies from 20 to 80. For each question, the score corresponding to the answer is assigned. For positive questions (1, 6, 7, 10, 13, 16, 19), the score was inverted (if the participant answered 4, the score is counted as 1). The higher the score, the greater the anxious behavior. Scores between 20 and 34 points indicate low trait anxiety; 35 to 49 points indicate moderate anxiety; scores between 50 and 64 points indicate high anxiety; and 65 to 80 points indicate very high anxiety.^30^

### Perceived Stress Scale – PSS 14

The questionnaire is based on the stress levels assessed at baseline.^31^ This instrument assesses the degree to which individuals perceive situations as stressful.

Participants answered this questionnaire alone, but help was available if they had any questions. The questionnaire consists of 14 questions with response options ranging from zero to four (0=never; 1=almost never; 2=sometimes; 3=almost always 4=always). Positive questions (4, 5, 6, 7, 9, 10 and 13) had their scores inverted as follows: 0=4, 1=3, 2=2, 3=1, and 4=0. Negative questions did not have their scores inverted.

The total was calculated using the sum of the scores of the 14 questions. The stress scores range from zero to 56. The higher the score, the greater the stress, thus, scores ≤ 18 indicate low stress; 19–24, normal stress; 25–29, moderate stress; 30–35, high stress; and > 35, very high stress.^31^

### Sample calculation

#### Sample power

Considering the mean initial standard deviation obtained in the two groups (4.62) and a 5% significance level with adjustment for a non-parametric procedure based on the relative asymptotic efficiency (n = n’/ 0.864), a sample of 20 subjects in each type of appliance has a power of 86% to detect a minimum difference of five compliance score units. The calculation was performed using the G*Power 3.1 (Heinrich Heine University, Düsseldorf, Germany).

### Interim analyses and stopping guidelines

Not applicable.

### Randomization

Simple randomization^32^ was performed by an external researcher using the Excel 2007 program (Microsoft Windows, Microsoft, Chicago, IL, USA), in a 1:1 proportion. The randomization codes were inserted in opaque, sealed, and numbered envelopes, consecutively, ensuring the concealment of the allocation into the two groups.

### Blinding

Patient and operator blinding was not possible in this study. However, the results were blindly analyzed. Code numbers were assigned to the patients by an independent researcher not involved in the outcome assessment.

### Statistical analysis

The t-test and the Fisher exact test were used to assess the compatibility between groups (age and gender). The internal consistency of the 10 questions of the scale was determined by the Cronbach alpha coefficient. Compliance comparisons between the two groups and between genders were performed using the Mann-Whitney test, and the comparison between the three times was done using the Friedman test. The correlation between age and compliance scores was verified by the Spearman correlation coefficient. In all tests, a significance level of p<0.05 was adopted and the SPSS version 27 program was used.

## Results

**Figure 2:**
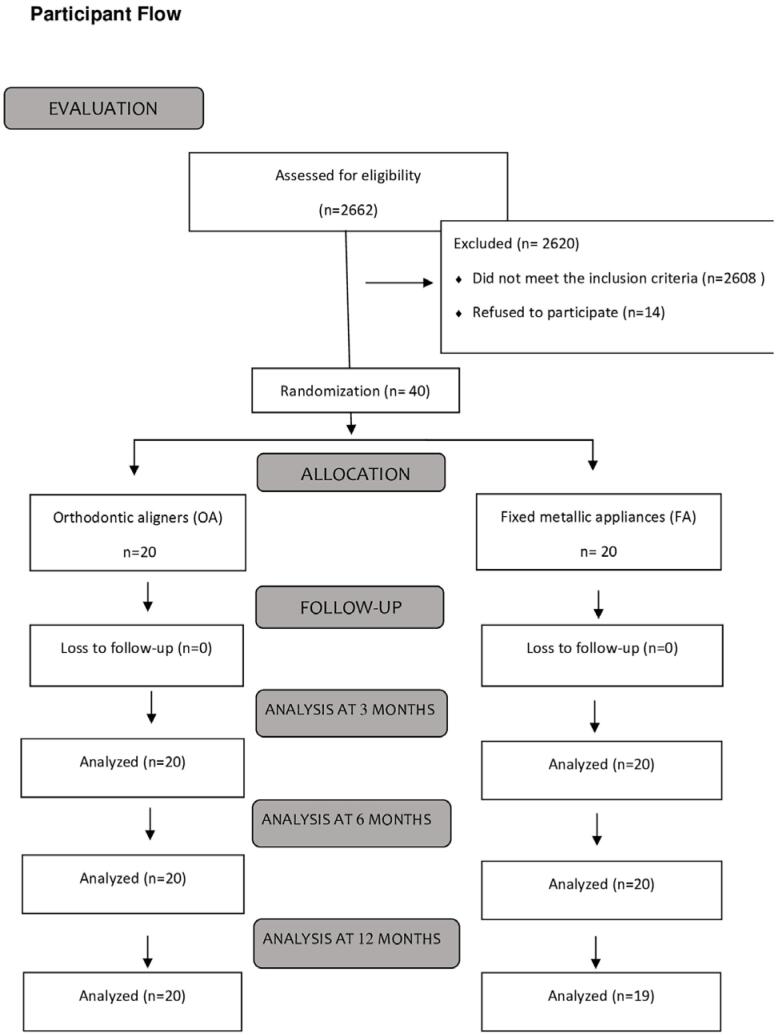
Consolidated Standards of Reporting Trials (CONSORT) diagram showing the patient flowchart throughout the trial.

Figure 2 shows the flowchart of patients assessed for eligibility for the study, randomized, allocated, and monitored in the first 12 months of treatment. Participants who met the inclusion criteria were recruited between August 2018 and February 2019. A total of 54 patients met the criteria and only 40 showed interest in receiving treatment. Orthodontic examinations were performed in February 2019. In May 2019, patients came for a post-randomization (baseline) appointment, appliance insertion and instructions. They returned once a month for monitoring over a period of 12 months.

The sample was composed of 14 (35.8%) females and 25 (64.2%) males. One patient in the fixed appliance group moved to another city after the sixth month of treatment. The mean age was 22 years, while the minimum age was 14 years and the maximum age was 35 years. The internal consistency of the questionnaire was measured by the alpha coefficient presented the results of 0.74, 0.79 and 0.95 in T1 (3 months), T2 (6 months), and T3 (12 months), respectively, and a value of 0.86 when the three times were combined.

### Baseline data

Participants of both groups were compatible regarding age, gender and psychosocial profile ( Table 1 ).

**Table 1:** Initial compatibility between groups regarding age, gender and psychosocial profile of patients.

Variable		Orthodontic aligner (n=20)		Fixed appliance (n=19)		p
Age (mean/SD)		23.7	5.6	20.5	4.5	0.053^ns^ †
Gender						
	Female (n/%)	8	40.0%	7	36.0%	1.000^ns^ ‡
	Male (n/%)	12	60.0%	12	64.0%	
Anxiety (mean/SD)		36.3	6.1	37.4	6.9	0.622^ns^ *
Stress (mean/SD)		19.4	6.9	21.8	7.4	0.302^ns^ *
Little's Irregularity Index		4.6	1.3	4.9	1.8	0.570^ns^ *

non-statistically significant difference

† - Paired t-test

‡ - Fisher exact test

* - Independent t-test

Table 2 shows the degree of patient compliance with the two orthodontic protocols: aligners and conventional fixed appliances, during the first 12 months of treatment. There was no difference in compliance between the two treatment protocols in the months evaluated.

**Table 2:** Comparison of compliance scores between appliances in the three evaluated times.

Time	Appliance	mean	sd	median	minimum	maximum	p
T1	OA	39.75	3.02	39.0	35	46	0.081 ns †
3 months	FA	36.95	5.51	37.0	21	45	
T2	OA	39.30	4.97	39.5	29	47	0.904 ns †
6 months	FA	38.80	5.36	40.0	21	45	
T3	OA	39.10	10.46	40.5	10	50	0.698 ns †
12 months	FA	38.85	7.65	41.0	21	47	

OA – Orthodontic Aligners; FA – Fixed Appliance

ns – non-statistically significant difference.

† - Mann-Whitney test

The comparison of patient compliance during the first year of treatment for each type of appliance was analyzed at 3 different times, as shown in Table 3 . No difference was found in the cooperation score between the third, the sixth and the twelfth months of treatment, regardless of the treatment protocol.

**Table 3:** Comparison of compliance scores between the three times, for each type of appliance.

Appliance	Time	mean	sd	median	minimum	maximum	p
OA	T1	39.75	3.02	39.0	35	46	0.396 ns †
	T2	39.30	4.97	39.5	29	47	
	T3	39.10	10.46	40.5	10	50	
FA	T1	36.95	5.51	37.0	21	45	0.164 ns †
	T2	38.80	5.36	40.0	21	45	
	T3	38.85	7.65	41.0	21	47	

OA – Orthodontic Aligners, FA – Fixed Appliance

ns – non-statistically significant difference

† - Friedman test

**Figure 3:**
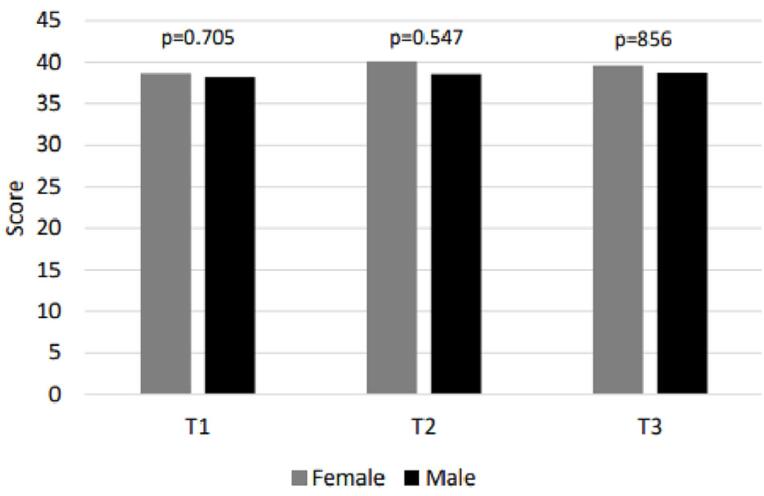
Mean female and male compliance scores at the three assessments.

There was no statistically significant difference between the degree of compliance and gender during the first year of treatment, as observed in Figure 3 .

### Adverse effects

The two appliances presented some minimal risk inherent to the orthodontic treatment, namely: slight shortening of the tooth root and mild discomfort after the monthly treatments. Serious harm was not observed.

## Discussion

This study compared the compliance of patients using orthodontic aligners and fixed appliances during the first year of treatment. The groups were initially compatible regarding age, gender and psychosocial profile, which eliminates the influence of these variables on the degree of patient compliance ( Table 1 ). The null hypothesis was accepted, indicating no statistically significant difference in patient compliance between aligners and fixed appliances during the initial 12 months of treatment.

Psychosocial factors, such as stress and anxiety, can influence the orthodontic patient's routine and may impact the patient's cooperation.^33 , 34^ Individuals in both groups were healthy young adults presenting normal levels of stress and a moderate level of anxiety ( Table 1 ). Patients who are anxious, stressed and/or poorly informed about the orthodontic treatment may fail to attend appointments and demonstrate poor compliance.^35^ Although a moderate level of anxiety was found, providing patients with detailed information during orthodontic treatment probably has a positive effect on motivation and prevents anxiety levels from negatively influencing patient compliance.^34^

The compliance of patients in the sample can be considered very good —with median compliance ranging from 37 to 41 points, and maximum compliance at 50. The degree of compliance was similar in the two treatment protocols. The use of aligners did not influence treatment compliance in the three periods evaluated ( Table 2 ). Timm, et al.^23^ (2021) evaluated the compliance of aligner patients and observed that 36% of the sample presented great compliance. Other studies found that the daily wear time of clear aligners ranges from acceptable to great.^6 , 36^

Table 3 shows that compliance remained stable during the first year of treatment. The scores were not significantly different between the third, sixth and twelfth months of treatment. Timm, et al.^23^ (2021) also did not find significant differences in adherence when evaluating aligner patients from the third to the twelfth months of treatment. The same was reported in a study applying the same scale (OPCS)^28^ to assess patients during orthodontic treatment with fixed appliances.^37^

Gender did not influence the degree of treatment compliance in the three periods evaluated ( Figure 3 ). Previous studies reported that men were significantly more compliant than women in the use of aligners,^23^ while women are more cooperative when using removable orthodontic appliances.^38^ Al-Abdallah, et al.^39^ (2021) found that female patients were more compliant with fixed orthodontic appliances. In this study, monthly follow-ups seem to minimize this difference between genders, which is in accordance with studies that did not find a significant link between gender and aligner daily wear time.^5 , 40^

The groups were compatible regarding age. Previous studies have shown no relationship between age and daily wear time.^41 , 42^ Timm, et al.^23^ (1980) found no association between age and adherence to aligner use in a wider age range of patient ages (18 to 64 years).

### Limitations

The level of compliance found may be influenced by the short follow-up time (12 months). A limitation of this study was the lack of follow-up after treatment completion. More studies with longer follow-up periods need to be conducted in the future.

Compliance was not measured in relation to patient perception or with the use of sensors to record wear time, though these methods could be applied in future research.

### Generalizability

Although this study was conducted in an academic setting where treatment was provided at no cost, patients demonstrated a high level of compliance. This may be attributed to the clinical research context, in which patient follow-up is more rigorous to ensure the reliability of the data collected. This factor should be considered when interpreting the results and generalizing the findings.

## Conclusion

The type of appliance, whether it is conventional fixed appliances or orthodontic aligners, did not influence the degree of patient compliance during treatment.

The degree of patient compliance remained stable during the first year of treatment, suggesting that treatment duration did not affect compliance.

Patient age and gender did not influence compliance scores with orthodontic treatment.

Data availability

The datasets generated and/or analyzed during the current study are available from the corresponding author upon reasonable request.
